# Fellowships, Grants, & Awards

**Published:** 2007-10

## Reducing Health Disparities Among Minority and Underserved Children (R21)

This initiative is designed to stimulate research that targets the reduction of health disparities among children. For purposes of this initiative, health disparities apply to children who have limited access to resources and privileges that affect their health. Thus, this initiative includes a focus on ethnic and racial minority children and populations of under-served children to include children from low-literacy, rural, and low-income populations, geographically isolated children, hearing- and visually impaired children, physically or mentally disabled children, children of migrant workers, children from immigrant and refugee families, and language minority children. Children are individuals 0–21 years of age. The primary purpose of this initiative, therefore, is to solicit intervention studies targeting one of the aforementioned groups. Rather than a singular approach, interventions using a multilevel approach (individual, health system, community, societal) are encouraged. In addition, basic studies designed to further delineate mechanisms/pathways of disparities that lead to the development of interventions are also encouraged. Specific targeted areas of research include biobehavioral studies that incorporate multiple factors that influence child health disparities such as biological (e.g., genetics, cellular, organ systems), lifestyle factors, environmental (physical and family environments), social (e.g., peer influences), economic, institutional, and cultural and family influences; studies that target the specific health promotion needs of children with a known illness and/or disability; and studies that test and evaluate the cost-effectiveness of health promotion interventions conducted in nontraditional settings. Strategic Plans on Reducing Health Disparities are located at http://www.ninr.nih.gov/AboutNINR/NINRMissionandStrategicPlan/, http://www.nhlbi.nih.gov/strategicplan/index.htm, http://pubs.niaaa.nih.gov/publications/StrategicPlan/NIAAASTRATEGICPLAN.htm, http://www.nichd.nih.gov, http://strategicplan.nci.nih.gov, and http://www.niams.nih.gov/an/stratplan/index.htm.

Chronic diseases (e.g., high blood pressure and diabetes) can disproportionately affect racial and ethnic minorities, including individuals from lower socioeconomic classes, women and children, and may affect these individuals' ability to attain and maintain health. Children are especially vulnerable and often have multiple risk factors for poor health. Recent U.S. census data show that 16.7% of children live in poverty and there are more poor children than in any other segment of population. Moreover, the population of children is becoming increasingly more ethnically and racially diverse and rates of uninsured health insurance are among the highest for these groups. Uninsured rates are reported to range from approximately 12% for Asian children to 21% for Hispanic children. The association between poverty, health status, race, ethnicity, insurance status, and access to good quality of health care, or any health care, is well documented. For example, the infant mortality rate for African-American infants is 2.5 times higher than for white infants. Black and Hispanic children are less likely to receive preventive services including dental care, emotional counseling and diet management; and poverty and minority race/ethnicity are associated with increased risk for childhood chronic and disabling diseases. Hospitalization for asthma is 40–100% more likely for minority children than for other children.

Asthma is one of the most common chronic disorders in childhood, currently affecting an estimated 6.2 million children under 18 years of age. Minority children and children living in poverty have a greater burden from asthma compared with white nonpoor children, and the same children are less likely to receive adequate treatment and are less likely to have family or community support for their asthma management. Other chronic diseases are also becoming increasingly common among the nation’s children, but particularly so among minority and underserved children. Recent studies estimate the prevalence of childhood chronic conditions, requiring ongoing specialized care, to be between 10 and 20%.

Obesity is another example of the complex issue of health disparities among minority and underserved children. Although childhood obesity has been a longstanding public health problem, recent increases have raised the level to epidemic proportions among U.S. children. In a 2005 Institute of Medicine (IOM) report, it was estimated that approximately 9 million children over 6 years of age were overweight. Among children 6–19 years, 31% were at risk for overweight, and 16% were overweight (BMI > 95th percentile for age growth charts). These findings, based upon measured weight and height, reflect a substantial and alarming increase in childhood obesity over the past 2 decades. Most worrisome, however, is that the current increase is most prominent among non-Hispanic black and Mexican-American children, with the increase in these groups at > 10% over the last 2 decades. For example, for non-Hispanic black and Mexican-American adolescents, overweight prevalence increased from 13.4% to 23.6% and from 13.8% to 23.4%, respectively. Despite recommendations for a healthier diet, recent data show that the usual diet of today's children includes foods high in saturated fat, high in calorie-dense foods, and low in fruit and vegetable consumption.

Poor dietary patterns and sedentary behaviors are linked not only to obesity but to the development of a number of seriously disabling and life- threatening conditions associated with obesity. For example, a potential complication of obesity is type II diabetes mellitus, a condition also increasing by epidemic proportions. Other complications likely to follow type II diabetes include increases in cardiovascular disease, kidney failure, and blindness. Besides type II diabetes, overweight and obese children and adolescents are at risk of becoming overweight adults with problems of coronary artery disease, hypertension, stroke, respiratory problems, gallbladder disease, osteoarthritis, sleep apnea, some forms of cancer, and premature death. These are serious health consequences facing minority and under-served children and families across the nation, in addition to the socioeconomic consequences such as suboptimal school performance, the potential for decreased productivity across the life course, social stigmatization, and high health care costs.

The landmark IOM Report (1999), although it made recommendations for a research agenda for the causes and solutions to health disparities, focused primarily on racial and ethnic disparities among adults. The more recent 2004 IOM report, *Children’s Health, the Nations’s Wealth: Assessing and Improving Child Health*, clearly identifies the need for more research on disparities among various groups of children.

By the year 2050, current census predictions are that racial and ethnic minority groups will comprise the majority of the U.S. population. This demographic is also predicted to change at a more rapid rate in the pediatric population. Thus, there is an urgent need for research studies focused on the elimination or reduction of health disparities among children to thwart potentially significant burdens on the health care system, and society as a whole.

The following are potential areas of research related to this funding opportunity announcement. These examples are not listed in any priority order and are not to be viewed as exhaustive or an exclusive listing of potential areas. Applications should identify constructs to be measured, review the relevant theoretical literature, and clearly identify research aims and design, along with the strengths and limitations of the proposal. Outcome measures should have sound psychometric properties and should be age, literacy, and language appropriate. The cultural and socioeconomic status of participants should also be considered.

Suitable topics for research include, but are not limited to the following: 1) studies that incorporate multiple factors (at least two of the following factors) such as genetic, physiological, social, psychological, economic and demographic, environmental, and cultural factors believed to influence child health disparities; 2) development of sensitive biological and or behavioral markers to predict risk, disease course, and progression of disease; 3) interventions designed to reduce risk factors and exposures that lead to development of one or more poor health outcomes; 4) culturally and developmentally appropriate interventions that promote increased physical activity and healthier dietary intake or other health enhancing child health behaviors (e.g., reduction in TV viewing); 5) studies that employ economic incentives to promote health; 6) intervention studies targeting well-child care, preventive care, or developmental (early interventional or rehabilitative) care; 7) intervention studies to prevent, delay, treat, or manage risk for disease or progression of disease due to altered physiological, behavioral, or physical status secondary to complications of pregnancy and or intrauterine exposures; 8) interventions that target the specific health promotion needs of children with a known illness and/or disability; 9) studies that test and evaluate the cost-effectiveness of health promotion interventions conducted in nontraditional settings; 10) studies that evaluate how gender, health literacy, and immigrant status (including legal and visa status) affect children’s health and access to health care; 11) studies of racism and racial discrimination as well as other forms of discrimination and their impact on children and caregiver experiences, health and access to health care; 12) studies of racial socialization, children’s emerging ethnic identity and gender roles and their impact on health behaviors and health outcomes; 13) intervention studies that target children and parent health beliefs, health literacy and the influence of peers and culture on health behaviors, health care utilization and health outcomes; 14) culturally sensitive intervention studies targeting patient–provider respect, communication, interactions and trust in relationships, health care utilization, adherence with treatment plans, and health outcomes; 15) development of innovative age and culturally appropriate biobehavioral measures/tools to assess risk for less optimal cognitive, behavioral, psychosocial, and physical functioning; 16) interventions that include technology in biomedical imaging and bioengineering that reduce health disparities, such as low-cost diagnostic imaging and point-of-care technologies; 17) interventions to identify and treat underserved; minority children at risk for adverse physical and mental health outcomes resulting from alcohol involvement such as *a*) children of parents or other caretakers impaired by alcohol disorders and who fail to access suitable and consistent health care for their children; *b*) children with fetal alcohol spectrum disorders (FASD) resulting from intrauterine alcohol exposure who need health care services equipped to provide early diagnosis and intervention; *c*) underserved adolescents already drinking or at high risk of initiating drinking; 18) studies exploring health disparities and cancers such as *a*) the influence of early life events on health outcomes and anthropometric variables across ethnic groups, including factors related to childhood and adult cancers; *b*) projects exploring the mechanisms of disease, including cancers, over the life course that explicitly address factors assessed over time from early in life through childhood and adolescence; *c*) factors (genetic, behavioral, etc.) increasing the risk of adverse long-term or late effects among ethnoculturally diverse or medically underserved children treated for pediatric cancer; *d*) energy balance interventions using a multilevel approach addressing overweight or obesity among survivors of childhood cancer; *e*) intervention studies that address the impact of attitudes, beliefs, experiences, or other relevant factors on health outcomes among survivors of childhood cancers; *f* ) culturally sensitive health promotion interventions that could ameliorate the risk of adverse outcomes related to cancer or its treatment.

This Funding Opportunity Announcement (FOA) will use the NIH Research Project Grant (R21) award mechanism.

The applicant will be solely responsible for planning, directing, and executing the proposed project.

This FOA uses “just-in-time” information concepts. It also uses the modular as well as the non-modular budget formats (see http://grants.nih.gov/grants/funding/modular/modular.htm).

Specifically, if you are a U.S. organization and are submitting an application with direct costs in each year of $250,000 or less (excluding consortium Facilities and Administrative [F & A] costs), use the PHS398 Modular Budget component provided in the SF424 (R & R) Application Package and SF424 (R&R) Application Guide (see specifically Section 5.4, “Modular Budget Component,” of the Application Guide).

U.S. applicants requesting more than $250,000 in annual direct costs and all foreign applicants must complete and submit budget requests using the Research & Related Budget component found in the application package for this FOA. See NOT-OD-06-096, 23 August 2006.

Applicants must download the SF424 (R&R) application forms and SF424 (R&R) Application Guide for this FOA through Grants.gov/Apply.

Note: Only the forms package directly attached to a specific FOA can be used. You will not be able to use any other SF424 (R&R) forms (e.g., sample forms, forms from another FOA), although some of the "Attachment" files may be useable for more than one FOA.

For further assistance, contact GrantsInfo, 301-435-0714, (telecommunications for the hearing impaired: TTY 301-451-0088) or by e-mail: GrantsInfo@nih.gov.

The application submission dates for this PA are available at http://grants.nih.gov/grants/funding/submissionschedule.htm.

The complete version of the PA is available at http://grants.nih.gov/grants/guide/pa-files/PA-07-391.html.

Contacts: The complete of agency contracts is available at http://grants.nih.gov/grants/guide/pa-files/PA-07-391.

Reference: PA-07-391

## International Research Ethics Education and Curriculum Development Award (R25)

Few developing country institutions provide formal education in research ethics, and few developed country programs for advanced research ethics education/training focus in depth on the internationally relevant aspects of research ethics. Therefore, few developing country scientists and health professionals conducting clinical or public health research have received extensive education and training in the principles of research ethics, international codes and legal aspects of ethical research, informed consent, elements of study design that affect the ethical conduct of research and the ethical framework for provision of care and risk/benefit analysis for study participants. To address this need, the Fogarty International Center (FIC) invites applications for International Research Ethics Education and Curriculum Development Program Awards to develop masters level curricula and provide educational opportunities for developing country academics, researchers, and health professionals in international ethics, related to performing research involving human subjects in international resource-poor settings. Alternatively, developing-country applicants may submit proposals to support program planning activities in preparation to apply for comprehensive program support in the future.

The goal of this initiative is to increase the cadre of developing country scientists, health professionals, and relevant academics with in-depth knowledge of the ethical considerations, concepts, and applications in clinical and public health research. It is expected that such advanced education/training will enhance the career development of individuals from developing countries, as well as strengthen expertise to support ethical clinical and public health research at their home institutions.

Proposed degree or nondegree comprehensive international research ethics education programs should equip academics, health professionals, and researchers from developing countries with the critical skills that are needed to subsequently provide research ethics education, ethical review leadership, and expert consultation to their institutions, national governments, and international bodies and, potentially, to pursue studies of ethical practice in clinical and public health research in developing countries.

Proposed curricula should provide a core set of advanced study courses that primarily focus on the internationally relevant aspects of ethical, legal, and moral principles guiding the responsible conduct of research. Appropriate educational activities may include practicum experiences, such as participation in ethical review committees, development of research ethics education/training courses for researchers and ethical review committee members at their home institutions, analysis of ethical review guidelines or processes and research on ethical practices in biomedical or behavioral research in the participants’ countries. Education may also be provided in areas such as research design methodology, technical manuscript and grant writing, statistical methods, informatics, and English as a second language, if needed. Curriculum developed in new comprehensive programs must be offered to participants after a maximum of 1 year of the award and should be ongoing in previously supported programs.

Planning grant proposals should describe in detail how curriculum components and educational activities for a comprehensive program will be designed during the 2-year award period.

Four-year comprehensive training program applications should propose degree or nondegree masters-level programs, including international research ethics curriculum and practicum experience for up to 2 years and no less than 12 months for developing country participants at the grantee, consortium, or home country institutions. Support can be provided for educating and training developing country academics such as ethicists or philosophers, researchers, and health professionals working at institutions conducting clinical or public health research.

New applications proposing research ethics education programs for participants from Francophone and Lusophone African countries, India, China, Thailand, Latin America, Russia, and Eastern Europe are especially encouraged due to the large amount of NIH supported research currently conducted in these countries.

This FOA will use the NIH Research Education Grant (R25) award mechanism. As an applicant, you will be solely responsible for planning, directing, and executing the proposed project. The applicant organization’s administration must provide the necessary management for the transfer of funds and materials to the collaborators and any subcontracts.

This FOA uses just-in-time concepts. It also uses the nonmodular budget format. Applicants must complete and submit budget requests using the SF424 Research and Related (R&R) Budget Component found in the application package for this FOA. A detailed categorical budget for the “Initial Budget Period” and the “Entire Proposed Period of Support” is to be submitted with the application.

Applicants must download the SF424 (R&R) application forms and SF424 (R&R) Application Guide for this FOA through Grants.gov/Apply.

Note: Only the forms package directly attached to a specific FOA can be used. You will not be able to use any other SF424 (R&R) forms (e.g., sample forms, forms from another FOA), although some of the "Attachment" files may be useable for more than one FOA.

For further assistance, contact GrantsInfo: 301-435-0714, (telecommunications for the hearing impaired: TTY 301-451-0088) or by e-mail: GrantsInfo@nih.gov.

The letter of intent date receipt date for this RFA is 14 November 2007, with the application receipt date 14 December 2007. The complete version of this RFA is available at http://grants.nih.gov/grants/guide/rfa-files/RFA-TW-08-002.

Contact: Barbara Sina, Division of International Training and Research, Fogarty International Center, Building 31, Room B2C39, Bethesda, MD 20892-2220 USA, 301-402-9467, fax: 301-402-0779, e-mail: sinab@mail.nih.gov.

Reference: RFA-TW-08-002.

